# Supplementary material for: Assessing the impact of a mandatory calorie labelling policy in out-of-home food outlets in England on consumer behaviour: a natural experimental study
Source: BMC Public Health. 2025 Mar 11;25:955. doi: 10.1186/s12889-025-22150-3 (PMC11895149; doi:10.1186/s12889-025-22150-3)
Supplement: Supplementary file 1 — Supplementary Material 1 [file 12889_2025_22150_MOESM1_ESM.docx]

**Supplementary Fig 1a-1f.** Marginal probability (%) of (a) noticed nutrition information, (b) used nutrition information, (c) ordered something different, (d) ate less of order, (e) changed restaurants visited, and (f) ate at restaurants less often from 2019-2022 for England, No Policy, and Non-England UK.
